# Assessing vaccine introduction and uptake timelines in Gavi-supported countries: are introduction timelines accelerating across vaccine delivery platforms?

**DOI:** 10.1136/bmjgh-2021-005032

**Published:** 2021-05-27

**Authors:** Karuna Luthra, Anna Zimmermann Jin, Prarthana Vasudevan, Karen Kirk, Carol Marzetta, Lois Privor-Dumm

**Affiliations:** 1 International Vaccine Access Center, Johns Hopkins University Bloomberg School of Public Health, Baltimore, Maryland, USA; 2 Vaccines & Sustainability, GAVI Alliance, Geneva, Geneva, Switzerland; 3 (Formerly Applied Strategies), Strategic Decisions Group, Palo Alto, California, USA; 4 Skoll Foundation, Palo Alto, California, USA; 5 Center for Health Security, Johns Hopkins University Bloomberg School of Public Health, Baltimore, Maryland, USA; 6 ESS, Cherry Hill, New Jersey, USA; 7 International Health, Johns Hopkins University Bloomberg School of Public Health, Baltimore, Maryland, USA

**Keywords:** immunisation, vaccines, health policy, health systems

## Abstract

**Background:**

Previous studies identified factors influencing regulatory approval to introduction timelines for individual vaccines. However, introduction and uptake timelines have not been comprehensively assessed across the portfolio of Gavi-supported vaccines.

**Methods:**

We analysed median times between introduction milestones from vaccine licensure to country introduction and uptake across six vaccine-preventable diseases (VPDs), three delivery platforms and 69 Gavi-supported countries. Data were gathered from public, partner and manufacturer records. VPDs and prequalified vaccines analysed included *Haemophilus influenzae* type b (DTwP-HepB-Hib, pentavalent), pneumococcal disease (pneumococcal conjugate vaccine, PCV), rotavirus diarrhoea (rotavirus vaccine, RVV), cervical cancer (human papillomavirus vaccine, HPV), polio (inactivated polio vaccine, IPV) and meningococcal meningitis (meningococcal group A conjugate vaccine, MenA).

**Results:**

Median time from first vaccine licensure to first Gavi-supported country introduction across VPDs at a ‘global level’ (Gavi-supported countries) was 5.4 years. Once licensed, MenA vaccines reached first introduction fastest (campaign=0.6 years; routine immunisation (RI)=1.7 years). Most introductions were delayed. Country uptake following first introduction was accelerated for more recently Gavi-supported RI vaccines compared with older ones.

**Conclusion:**

Factors accelerating timelines across delivery platforms included rapid product prequalifications by WHO, strong initial recommendations by the WHO Strategic Advisory Group of Experts (SAGE) on Immunization, achieving target product profiles on first vaccine licensure within a VPD and completing several VPD milestones at a global level prior to licensure. Milestones required for introduction in Gavi-supported countries should start prior or in parallel to licensure to accelerate uptake of vaccines delivered through diverse delivery platforms.

**Key questions**

**What is already known?**

- Most of the existing peer-reviewed literature on new vaccine introduction (NVIs) and regulatory timelines are from high-income settings, such as Europe.
- Some information is available on the timelines for individual vaccines for routine immunisation and selected portions of the NVI process in low-and-middle-income countries (LMICs), often outlined as lessons learned.
- A 2016 study by Ahonkhai *et al* examined regulatory timelines for market access and WHO prequalification in LMICs.

**What are the new findings?**

- This study is the first to examine NVI and uptake timelines across multiple Gavi-supported vaccines and delivery platforms, over the past two decades, and quantifies ‘global-level’ vaccine-preventable disease (VPD) NVI milestones along the full pathway from first licensure through first country introduction and uptake across the Gavi cohort, tracking ‘country level’ NVI milestones of Gavi funding application submission through target coverage in the majority of Gavi-supported countries.
- Study findings illuminate the diversity in NVI and uptake timelines, which differ by VPD and delivery platform, including RI, campaign and adolescent delivery platforms.
- Timelines for new RI vaccines have accelerated over time, partly due to planned global efforts by partners at all levels to accelerate introductions and uptake.

**Key questions**

**What do the new findings imply?**

- Opportunities exist to improve NVI timelines, particularly if multiple global-level and country-level steps are completed prior to vaccine licensure.
- This study provides a starting place against which to benchmark timelines for NVIs for new vaccines, particularly for RI and traditional campaign vaccines.
- Further understanding and measurement of how to accelerate NVI timelines is needed across all delivery platforms, with new lessons to be learned from establishing new delivery platforms and reaching broader populations with COVID-19 vaccines.

## Introduction

Low-and-middle-income countries (LMICs) historically adopted vaccines more slowly than high-income countries.^1–3^ Accordingly, Gavi, the Vaccine Alliance, was created in 2000 to bring together UN agencies, governments, donor countries and the private sector to accelerate vaccine access in LMICs. From 2000 to 2014, Gavi catalysed the introduction of 11 new and underused vaccines across diverse vaccine delivery platforms (eg, routine infant immunisation (RI) programmes, immunisation campaigns and adolescent immunisation programmes) in 73 Gavi-supported countries, resulting in an additional 500 million children immunised.^4^

However, despite progress by Gavi, its partners, and Gavi-supported countries to expand and strengthen national immunisation programmes, and global targets for universal vaccine access by 2020, disparities persist in the number of new vaccine introductions (NVIs) planned or achieved in LMICs, with one in five children globally still not receiving their standard set of infant immunisations.^1 5^ Benchmarks of NVI introduction and uptake timelines in Gavi-supported countries have not been comprehensively quantified and communicated, as they have been in high-income nations, and reporting of vaccine access and introduction timelines in LMICs has been fragmented.^6 7^ For example, studies covering Europe and LMICs report on factors influencing individual vaccine access and the amount of time from regulatory approval to introduction for a few individual RI vaccines, including analyses covering hepatitis B vaccine, *Haemophilus influenzae* type b (Hib) vaccine, pneumococcal conjugate vaccine (PCV), rotavirus vaccine (RVV) and human papillomavirus vaccine (HPV), as well as factors influencing these individual vaccine timelines.^7 8^ Timelines for WHO prequalification (PQ) in LMICs have also been reported, but uptake by countries after product registration was not examined.^9^ In addition, no comprehensive assessment of NVI timelines across Gavi’s portfolio or of the drivers and barriers that could help accelerate NVI and uptake in LMICs has been done.

Assessing the pace of Gavi-supported NVIs can help identify ways to accelerate increasingly diverse future introductions across multiple delivery platforms. In the context of COVID-19, understanding previous bottlenecks or areas of acceleration combined with lessons learned from vaccines that had intense global support, new delivery platforms and target populations provides benchmarks for what timelines could be targeted and feasible for future NVIs.

The objective of this study is to analyse NVI and uptake timelines over the past two decades across selected Gavi-supported vaccines spanning different delivery platforms for the entire cohort of Gavi-supported countries. It hypothesises a standard NVI milestone pathway from vaccine licensure to country uptake, and aims to describe the milestone pathways to NVI and uptake (if different than hypothesised) and common factors associated with delays or acceleration across different VPDs and diverse vaccine delivery platforms. We also aim to illuminate cross-platform insights and enable understanding of the broad drivers and barriers to accelerated vaccine introductions. We also sought to establish baseline timelines that could provide NVI timing benchmarks across diverse vaccine delivery platforms for existing Gavi-supported vaccines and for new vaccines that may be supported by Gavi in the future.

## Methods

We hypothesised a standard pathway from vaccine licensure to NVI and uptake in Gavi-supported countries, based on previous experience and discussions with NVI experts. We measured timelines between milestones for six VPDs, 38 vaccines (listed in online supplemental table 1) and 69 Gavi-supported countries (listed in online supplemental table 2).^4^ Results are presented by individual VPD at a global level, across all VPDs at a global level and across Gavi-supported countries.

10.1136/bmjgh-2021-005032.supp1 Supplementary data

### VPD selection

Six VPDs were selected to represent diverse vaccine delivery platforms, including RI, campaigns and adolescent immunisation programmes. The VPDs and associated Gavi-supported vaccines were *Haemophilus influenzae* type b disease (DTwP-HepB-Hib, pentavalent vaccine), pneumococcal disease (PCV), rotavirus diarrhoea (RVV), cervical cancer (HPV), polio (inactivated polio vaccine, IPV) and meningococcal meningitis (meningococcal group A conjugate vaccine, MenA). Where deemed substantively different, PCV results are reported separately for all PCV products and only PCV10/PCV13. For VPD milestones at the global level, data from HPV demonstration projects (demo) are included, while at the country level HPV results are split into demo and RI. MenA results are reported separately across different delivery platforms (RI for infants and campaigns for 1 to 29 years of age). IPV is only included in analysis of country-level milestones and not the global-level VPD milestones. It is considered an outlier given the long gap in time from first licensure (1982) to SAGE recommendation (2004) and was not considered comparable with other vaccines included in the study, which did not experience such a long gap.^10^

### Vaccine introduction milestone data

An NVI milestone was defined as a required step in the process for Gavi-supported introductions to take place. These included milestones spanning from the first vaccine licensure (any country) to NVI and uptake in a Gavi-supported country. Milestones were categorised as ‘global-level’ VPD milestones if they were a major step taken by global partners, such as public health institutions, vaccine manufacturers and implementation agencies necessary for a Gavi-supported introduction to take place (eg, SAGE recommendations or WHO PQ licensure), but were not tied to a specific country. Milestones were categorised as country-level milestones if they were a major step taken by Gavi or Gavi-supported countries that were tied to a specific country’s NVI process, beginning with submission of the country’s funding application for Gavi support through the Gavi-supported NVI and target coverage level reached. The full list of global-level VPD and country-level milestones is available in online supplemental table 3. Dates were collected from public sources and from partners’ internal records through December 2017.

As reported in online supplemental table 3, data were sourced from various public and private records. Global-level VPD milestones were gathered from WHO records,^11^ industry records,^12^ regulatory agencies’ records,^13–24^ SAGE meeting records^25^ as well as private data provided through personal communications by international organisations and initiatives (Gavi, the Vaccine Alliance, 2016 and 2018; WHO, 2016; UNICEF, 2016; the Hib Initiative, 2016) and vaccine manufacturers (Johnson & Johnson, 2016; Biological E Limited, 2016; Pfizer, 2016). Country-level milestones were gathered from private data provided via personal communications from partners (Gavi, the Vaccine Alliance, 2016 and 2018 and Gavi’s public records).^26 27^ Vaccine coverage rates from 1998 through 2016 for 69 Gavi-supported countries were retrieved for the RI vaccines analysed (pentavalent, PCV, RVV, IPV) and the third dose of diphtheria-tetanus-pertussis vaccine (DTP3) and were sourced from WHO and UN databases.^28 29^ All milestone data were available at the global VPD level; however, some milestone data were missing at the country level. Where milestone data were missing or a country had not yet completed that milestone, we excluded that data point from the analysis and noted the sample size for each milestone included in the analysis.

### Analysis of vaccine introduction and uptake

The NVI pathway was modelled as the expected chronological order of milestones that all VPDs at a global level would take following first licensure, regardless of delivery platform (online supplemental figure 1). The time between consecutive and selected combinations of important non-consecutive milestones was calculated. The median time between milestones was calculated across VPDs at a global level, individual VPDs at the global level and across all countries at the country level.

At the global VPD level, we calculated medians for both the ‘expected’ and chronological pathways and analysed if specific sequences of milestones influenced the pace of NVIs. At the country level, we included data from all country introductions. This included a country’s first NVI date regardless of funder to assess overall country performance.

To analyse the pace of uptake, we compared each country’s annual vaccine coverage rates for up to four RI vaccines (pentavalent, PCV, RVV, IPV) against the country’s DTP3 coverage rates for the same year. We used individual country-level DTP3 rates as the target coverage to describe a minimum of vaccine performance to achieve for an NVI. DTP3 coverage is a widely accepted indicator of RI system performance, and one indicator for Gavi’s vaccine goal during its 2016–2020 strategic period.^10 30^ Although other targets could have been selected, we chose this as a reasonable baseline reflecting individual country performance. The target is not intended to describe optimal potential performance. We assessed both the number of countries and the percentage of the ‘Gavi-cohort’ (ie, total number of surviving infants across 69 Gavi-supported countries) achieving 50% and 100% of the target coverage rate.^10 11^ Given considerable variability in the data quality of national vaccine coverage estimates and to account for potential discrepancies in coverage reporting, target coverage was considered achieved once a country was within 10% of the country’s own DTP3 rate.

Additional desk research and input from experts and partners helped illuminate key drivers and barriers behind different timelines.

### Ethical considerations

This study was presented to the Johns Hopkins Bloomberg School of Public Health IRB and granted IRB exemption as a public health practice research since information sought was about institutions and countries, not individuals. No patients were involved in the conduct of this study.

## Results

### Global pathways from first licensure to first country introduction

**Figure 1:**
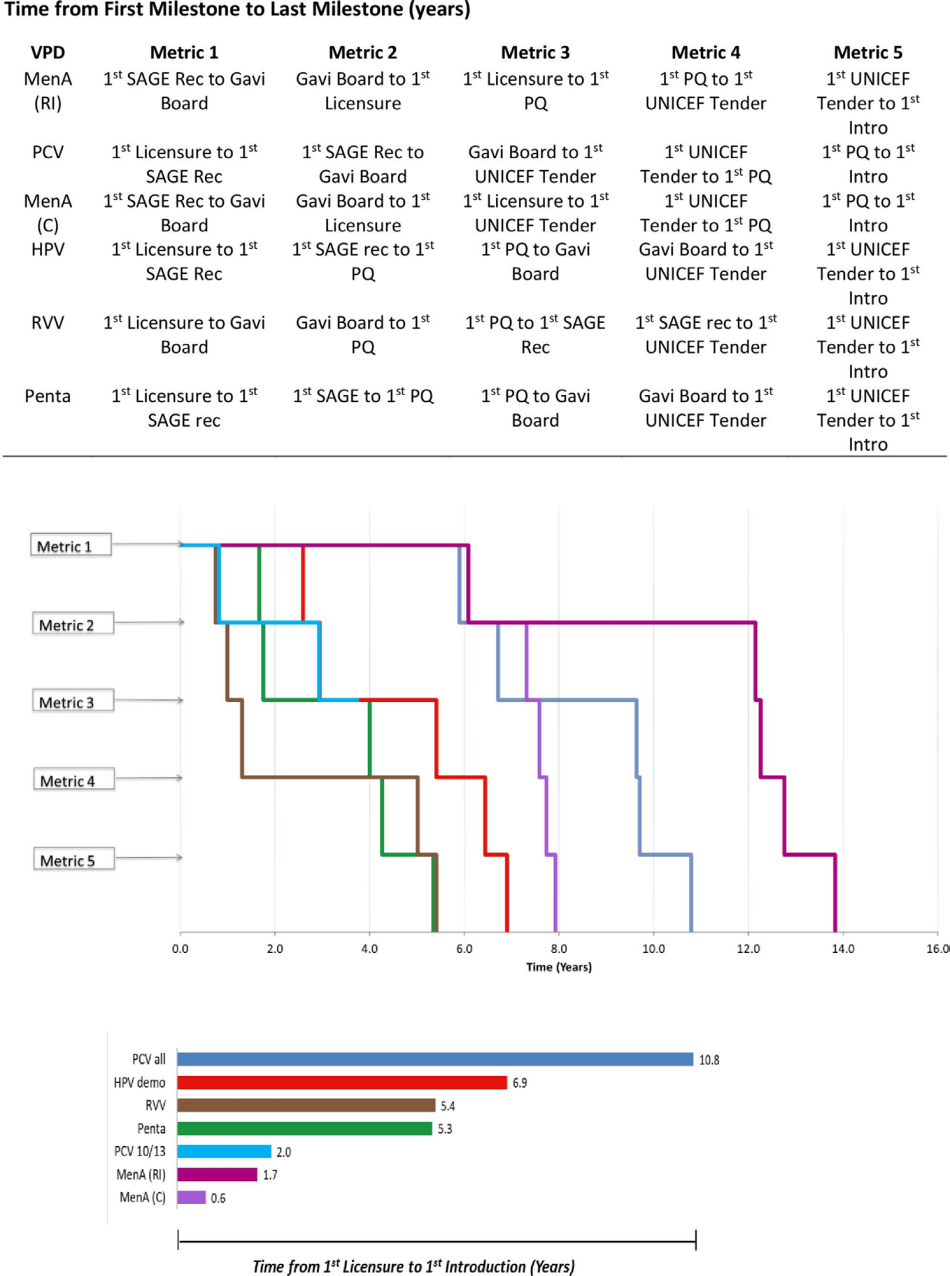
Chronological milestone order and times between milestones for all global-level VPDs, and time from first global-level VPD milestone to first country introduction for all VPDs. Licensure=1st WHO-approved NRA licensure per VPD; SAGE=1st SAGE recommendation; Gavi=Gavi Board approval; PQ=1st WHO prequalified vaccine by VPD; UNICEF=Unicef tender issued; Intro=1st Gavi-supported country introduction; excludes time to target coverage (DTP3) rate reached because only Pentavalent and PCV reached 50% of target coverage (DTP3) rate milestone. Abbreviations: HPV, human papillomavirus vaccine; MenA, meningococcal group A conjugate vaccine; NRA, national regulatory authority; PCV, pneumococcal conjugate vaccine; Penta, pentavalent vaccine; RVV, rotavirus vaccine; SAGE, WHO Strategic Advisory Group of Experts on Immunization; VPD, vaccine-preventable disease.

Median time from first product licensure to first Gavi-supported introduction across the VPDs analysed at the global level was 5.4 years (n=6, range 0.6–10.8 years) (table 1). While all VPDs analysed completed each global milestone through introduction, no VPD analysed followed the exact hypothesised sequence for global-level VPD milestones (figure 1; online supplemental table 5A). Most introduction pathways began with first licensure with the exceptions of MenA (campaign), MenA (RI) and PCV10/PCV13, where a SAGE recommendation was the earliest milestone. The median time to first Gavi-supported country introduction across all global-level VPDs increased to 7.4 years (n=6, range 5.3–13.8 years) when considered from first milestone (online supplemental figure 2).

**Table 1:** Summary of median time between two milestones calculated across all VPDs and Gavi-supported countries

Starting milestone	Ending milestone	Median time (range), years*	IQR, years	N†
**Global-level VPRs**				
1st milestone per VPD‡	1st Gavi-supported country introduction	7.4 (5.3, 13.8)	5.8–10.1	6
1st NRA licensure per VPD	1st Gavi-supported country introduction	5.4 (0.6, 10.8)	2.6–6.5	6
1st WHO PQ per VPD	1st Gavi-supported country introduction	2.6 (0.2, 4.4)	1.2–3.9	6
1st SAGE Recommendation per VPD	1st Gavi-supported country introduction	4.6 (3.7, 13.8)	4.2–7.2	6
Gavi Board approval	1st Gavi-supported country introduction	3.0 (1.3, 7.7)	1.8–4.7	6
1st Gavi-supported country introduction	50% of target coverage§ across Gavi cohort	6.1 (2.3, 8.2)	4.2–7.1	3,¶**
50% of target coverage§ across Gavi cohort	100% of target coverage§ across Gavi cohort	7.0	–	1¶**
Gavi Board approval	Gavi application window opened	0.5 (−0.2, 6.2)	0.1–0.7	6
Gavi application window opened	1stGavi-supported country introduction	1.5 (0.6–4.0)	1.3–2.6	7**
**Country-level milestones**				
Country application submitted	Country application approved	0.4 (−0.3, 3.7)	0.3–0.7	251
Country application approved	Vaccine introduction in country	1.1 (−1.4, 5.0)	0.8–1.6	233
Vaccine introduction in country	Target coverage§ reached in country	1.4 (−10.1¶, 5.1)	1.1–1.9	162‡¶
Country application submitted	Vaccine introduction in country	1.6 (−1.4, 7.1)	1.2–2.5	291
Country application submitted	Target coverage§ reached in country	3.3 (−9.8††, 8.2)	2.6–4.6	153
Gavi application window opened	Country application submitted	2.2 (0.0, 12.3)	0.6–4.9	334
Country application approved	VIG disbursed	0.4 (−6.4, 2.8)	0.2–1.0	223
VIG disbursed	Vaccine introduction in country	0.5 (−1.9, 7.0)	0.3–0.9	263
Projected country introduction	Vaccine introduction in country	0.5 (−0.7, 7.1)	0.1–1.2	282

*Times can be negative when the milestones occurred chronologically out of the specified order.

†N refers to the number of VPDs analysed at the global level and number of countries analysed at the country level.

‡First chronological milestone—often, but not always the same as the first expected milestone.

§Target coverage is defined as within 10% of the country’s own DTP3 rate. Target coverage rates will vary across countries.

¶IPV included in this global-level VPD milestone as it pertains to country-level data.

**Only pentavalent, PCV and IPV reached 50% target coverage across the entire Gavi cohort, and only pentavalent reached 100% target coverage across the entire Gavi cohort.

††Extreme negative outliers stem from a country reaching target vaccine coverage using a Hib-containing vaccine prior to switching to pentavalent vaccine.

IPV, inactivated polio vaccine; NRA, national regulatory authority (WHO recognised); PCV, pneumococcal conjugate vaccine; SAGE, WHO Strategic Advisory Group of Experts on Immunization; VIG, vaccine introduction grant; VPD, vaccine-preventable disease; WHO PQ, WHO prequalification.

### Global-level VPD timelines for ‘newer’ versus ‘older’ Gavi-supported vaccines

At the global level, the VPDs for which the Gavi Board approved support for vaccine programmes earlier in Gavi’s history (2000–2006, referred to as ‘older’ vaccines) (pentavalent, PCV, RVV) generally had slower timelines from first licensure to first Gavi-supported country introduction than those whose vaccine programmes were more recently approved by the Board (2008–2013, referred to as ‘newer’ vaccines) (MenA (campaign), MenA (RI)), with the exception of HPV (figure 1). Excluding PCV7, which was introduced only in two countries and without Gavi support, PCV10/PCV13 produced similar timelines to MenA (RI and campaign) (figure 1, online supplemental table 5B). Introduction timelines were particularly accelerated for VPDs that completed major global-level VPD milestones, such as first SAGE recommendation or Gavi Board approval, prior to first licensure (PCV10/PCV13, MenA (C), MenA (RI)), or when WHO PQ quickly followed first licensure (MenA (C), MenA (RI)) (online supplemental table 5A, B). Timelines from date of first product PQ within a global-level VPD to first Gavi-supported introduction were faster for VPDs that received first PQ more recently (MenA (RI) (1.6 years), MenA (C) (0.2 years)) than for VPDs that received first PQ less recently (pentavalent (3.6 years); RVV (4.4 years)) (online supplemental table 5B).

Timelines for vaccine product milestones across all VPDs are reported in online supplemental table 4.

### Country-level decision-making and introduction timelines

**Figure 2:**
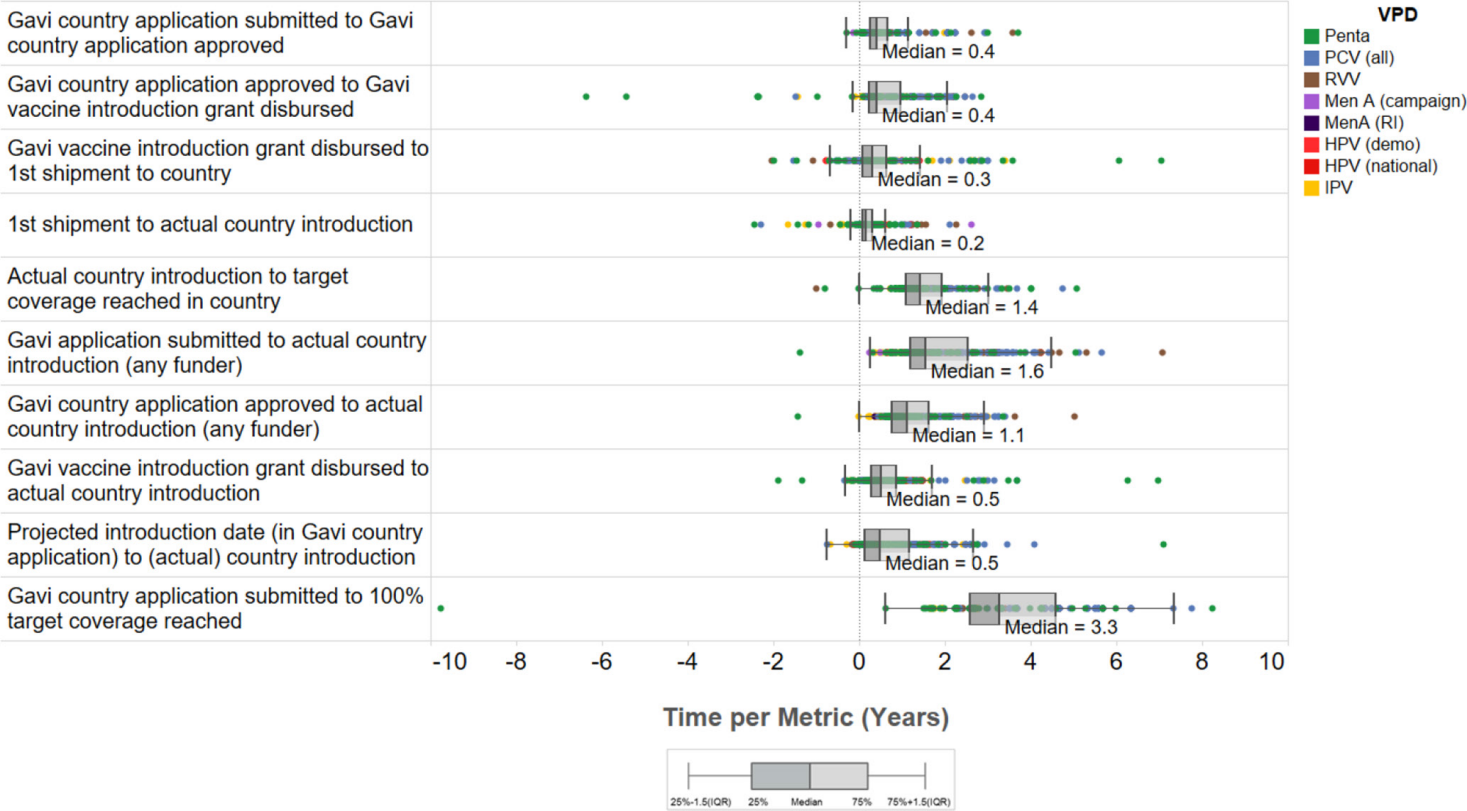
Country-level introduction milestone data across all country introductions and VPDs. Country-level introduction milestone data across country introductions of pentavalent, PCV (all), RVV, IPV, HPV (national), HPV (RI), MenA (C), MenA (RI) vaccines. Pentavalent and PCV experienced the greatest variation in introduction timelines across Gavi-supported countries. Abbreviations: HPV, human papillomavirus vaccine; IPV, inactivated polio vaccine; MenA, meningococcal group A conjugate vaccine; PCV, pneumococcal conjugate vaccine; RI, routine immunisation; RVV, rotavirus vaccine; VPD, vaccine-preventable disease.

At the country level, NVI implementation timelines also varied across VPDs. While Gavi application window openings generally quickly followed Gavi Board Approval to fund a new vaccine programme (median 0.5 years, range −0.2 to 6.2 years, n=6), the median time from the application window opening to the first Gavi-supported introduction in a Gavi-supported country across all VPDs was longer at 1.5 years (range 0.6–4.0 years, n=7) (table 1). Median time from a country submitting an NVI funding application to the country introducing a vaccine was 1.6 years (range −1.4 to 7.1 years, n=291) (figure 2). Following application approval, 56% of countries (n=233) waited one or more years to introduce, and 18% waited two or more years. At the country level, PCV (1.6 years, n=55) and RVV (1.7 years, n=36) had the longest median time to country introduction following application approval (online supplemental figure 3A).

**Figure 3:**
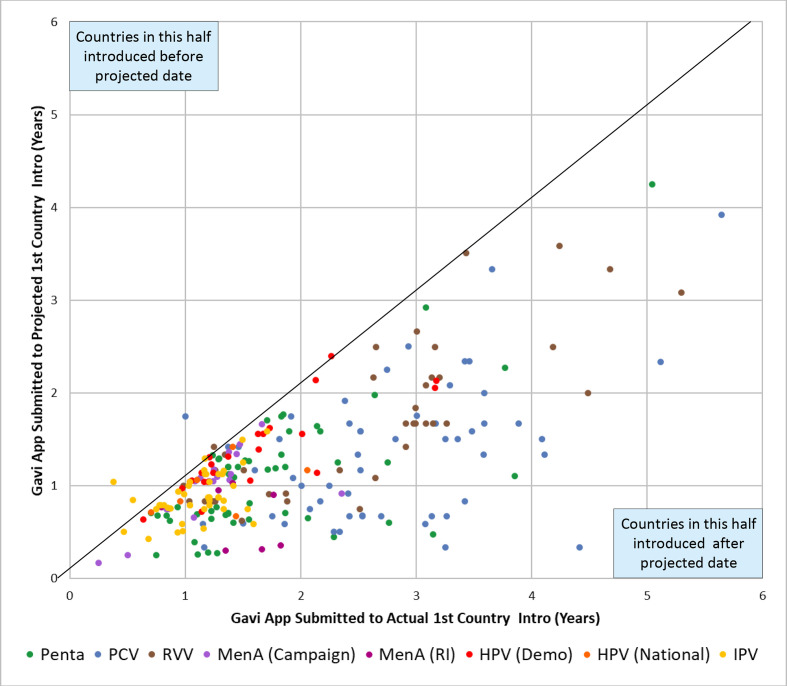
Projected introduction timelines compared with actual introduction timelines. Projected introduction timelines vs actual introduction timelines as stated in Gavi funding applications. The majority of countries introduced after their projected introduction date in the Gavi funding application across all VPDs. Abbreviations: HPV, human papillomavirus vaccine; IPV, inactivated polio vaccine; MenA, meningococcal group A conjugate vaccine; PCV, pneumococcal conjugate vaccine; Penta, pentavalent vaccine; RI, routine immunisation; RVV, rotavirus vaccine; VPD, vaccine-preventable disease.

Many countries experienced delays in their planned introductions and the median delay from planned to actual introduction date was 0.5 years (range −0.7 to 7.1 years, n=282), with the ‘older’ Gavi-supported vaccines (Penta, PCV, RVV) experiencing longer delays than the ‘newer’ Gavi-supported vaccines (MenA (C), MenA (RI), HPV (demo), HPV (RI)) (figure 3). Introduction delays of 1 year or longer occurred in 33% (n=261) of countries across all VPDs (online supplemental figure 3B).

The median timeline following the Gavi NVI funding application window opening to a country’s submission of the NVI funding application across all VPDs and countries was 2.2 years (n=334, range 0.0–12.3 years) (table 1). Country timelines for NVI funding application submission were not accelerated for the ‘newer’ Gavi-supported vaccines (HPV (national), MenA (C), MenA (RI)) compared with the ‘older’ Gavi-supported vaccines (Penta, PCV, RVV) (online supplemental table 6).

### Vaccine uptake for routine immunisations at country level

**Figure 4:**
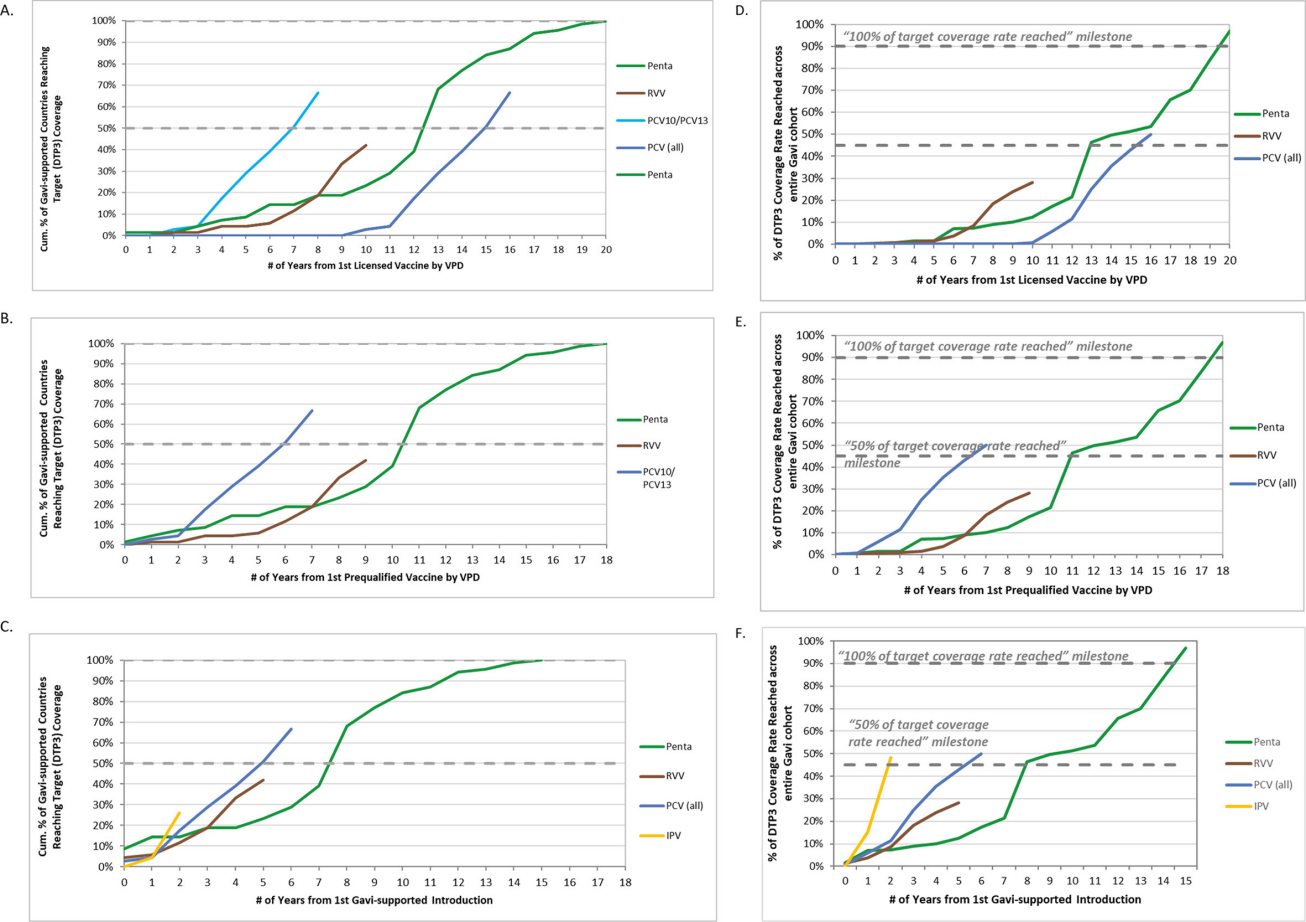
Progress towards reaching target coverage rates by routine immunisation vaccines. Cumulative percentage of Gavi countries reaching target coverage rate (ie, once a country is within 10% of the country’s own DTP3 rate) each year for pentavalent vaccines (Penta, Hib3 coverage), rotavirus vaccines (RVV, RVV last dose coverage), pneumococcal conjugate vaccines (PCV, PCV3 coverage) and inactivated polio vaccines (IPV, IPV first dose) across all Gavi countries from first licensed vaccine (A), first WHO prequalified vaccine (B) and from first Gavi-supported introduction (C). Progress in reaching target coverage rate for pentavalent vaccines (Penta, Hib3 coverage), rotavirus vaccines (RVV, RVV last dose coverage), pneumococcal conjugate vaccines (PCV, PCV3 coverage) and inactivated polio vaccines (IPV, IPV first dose) across the entire Gavi surviving infant cohort from first licensed vaccine (D), first WHO prequalified vaccine (E) and from first Gavi-supported introduction (F). IPV data are excluded from A, B, D and E given IPV was not included in global-level VPD analysis. PCV data separated out in A and D as the timeline for PCV10 and PCV13 vaccines differed from the timeline for PCV7.

Country uptake across the 69 Gavi-supported countries analysed also varied across VPDs. Among the RI vaccines, as of 2016, target coverage levels were reached by 69 countries (100%) for pentavalent (Hib3), 46 countries (67%) for PCV (PCV3), 29 countries (42%) for RVV (RVV last dose) and 18 countries (26%) for IPV (IPV1) (figure 4A–C). Across these four VPDs, the median time among countries that achieved target coverage was 1.4 years (range −10.1 to 5.1, n=162) (table 1) following introduction, with 78% (n=132) of these countries reaching target coverage in 2 years or less.

Across the entire cohort of children in Gavi-supported countries reached with DTP3, Gavi-supported pentavalent vaccine reached 97% (introduced in n=69 countries), PCV reached 50% (n=56 countries), RVV reached 28% (n=44 countries) and IPV reached 48% (n=53 countries) of these children, respectively, as of 2016 (note: coverage data were not available for all countries that had introduced). Pentavalent took 20.5 years from first NRA licensure, 18.8 years from first PQ and 15.2 years from first country introduction to reach target coverage across the entire Gavi cohort (figure 4D–F). PCV took 16.9 years from first NRA licensure (8.1 years if only PCV10/PCV13 are considered) and 6.1 years from first Gavi-supported introduction to 50% of target coverage across the Gavi cohort (figure 4A, C). IPV took only 2.3 years from first Gavi-supported introduction to reach 50% of target coverage across the Gavi cohort (figure 4F) and experienced the quickest uptake at the country level among the RI vaccines analysed (figure 4C).

## Discussion

This analysis presents NVI and uptake timelines that could provide timing benchmarks for existing Gavi-supported vaccines as well as for new vaccines that may be supported by Gavi in the future, many of which may span increasingly diverse delivery platforms. While the time from vaccine licensure to NVI in LMICs is widely understood to have shortened since the creation of Gavi, our analysis helps quantify this picture across a sub-set of diverse Gavi-supported vaccines and a range of delivery platforms. It also includes a deeper dive into the individual milestones completed than previous analyses that considered the time from licensure to uptake in high-income countries versus LMICs, including details about country-level milestones necessary for Gavi-supported introductions, coverage performance, and across several delivery platforms.

Our analysis highlights a median time frame from first licensure to first Gavi-supported NVI of approximately five and a half years. It also helps illuminate potential NVI timelines, which is particularly important in light of new vaccines, including COVID-19 vaccines, to both compare timelines against existing vaccines, and for non-pandemic vaccines to monitor if timelines shift given the current context. Some NVI timelines have been relatively short by historical standards (MenA (campaign and RI) and PCV10/PCV13, for example, took approximately 6 months to 2 years to complete first introduction, and IPV surpassed all other vaccines in the speed of application submissions and subsequent rate of uptake). Still, further monitoring is needed to determine if these should become standard timeframes, and if these timelines are impacted either positively or negatively by the pandemic. Coordinated efforts among the global vaccine community may be needed to ensure critical milestones for new vaccines are completed in parallel with or before first licensure, and that the first product licensed within a VPD meets the desired target product profile (TPP) for LMICs. In addition, a strong global push and dedicated global initiatives can help accelerate what happens at the country level, as seen with IPV. Nonetheless, multiple paths may be possible to achieve accelerated NVI outcomes, as illustrated in the variation of the order of milestones taken to reach first introduction.

We also saw that accelerated first introductions within a VPD were facilitated by completion of multiple global-level milestones prior to or in tandem with first licensure, primarily the first SAGE recommendation and Gavi Board approval for support. WHO PQ following very soon after licensure also accelerated overall timelines. At the country level, however, a subsequent ‘strong’ SAGE recommendation or several SAGE recommendations preceding first licensure rather than the initial recommendation at licensure helped facilitate faster country application submissions, such as for MenA (campaign), and PCV10/PCV13. In addition to SAGE recommendations applicable for all Gavi-supported countries, increased global attention was also important for accelerating applications, as was seen for pentavalent following the establishment of the Hib initiative and an updated SAGE recommendation.^31^

Along with elucidating factors conducive to accelerate or delay timelines at the global VPD level, our analysis also highlighted that NVI delays were experienced by countries across all vaccines regardless of delivery platforms or if a vaccine was ‘older’ or more recently supported by Gavi. However, we did find that the more recently supported Gavi vaccines experienced introduction delays shorter in duration than the ‘older’ Gavi vaccines. Delays have previously been attributed to supply constraints, which were exacerbated by lack of demand predictability and limited trust by manufacturers in Gavi’s strategic demand forecasts. PCV experienced the greatest number of delays, despite significant efforts such as the PneumoADIP and Gavi’s Advance Market Commitment, which helped improve the supply outlook and aimed to accelerate country adoption. Across other RI vaccines, efforts to accelerate NVI and address supply constraints of new vaccines also had varying degrees of success. Additional barriers to uptake of these vaccines may relate to concerns about the product itself including efficacy, and/or serotype coverage, as was the case for PCV7 versus PCV10/PCV13, and availability of sufficient cold chain space, such as for RVV. As shown in our analysis, timelines for PCV implementation are much shorter when only PCV10/PCV13 products were included, furthering the case for how achieving the desired TPP can potentially accelerate NVI timelines.

Among the VPDs, HPV was the only one analysed that required a new delivery platform to be established. This was also the only vaccine included in this study not supported through a dedicated global initiative during the time period covered. Compared with the RI and campaign vaccines analysed, time to first HPV introduction was slow; only half of eligible countries submitted applications for demonstration projects, and far fewer had applied for introduction into RI systems at the time of data collection. It is not clear, however, given both the mixed timelines of the other vaccines and the requirement at the time for countries to first implement demonstration projects, whether HPV timelines would be different had a dedicated global initiative existed, particularly given the higher cost of the vaccine, health systems complexities for vaccines requiring new delivery strategies and platforms and significantly limited global supply available to Gavi-supported countries, particularly following the expansion of Gavi’s HPV program in 2016.^32^ Considering new products in the pipeline from lower cost manufacturers and several new studies on the efficacy of a one-dose regimen instead of the SAGE recommended two or three doses, HPV may be an important analogue to study further, particularly for maternal immunisation or vaccines targeting populations outside of infants, as well as for other products that may experience significant supply-side challenges.^33^

As countries continue to introduce new vaccines, the challenge shifts from completing all country introductions to accelerating the pace of NVIs and coverage levels achieved following introduction. The pace of additional countries introducing new vaccines and achieving target coverage rates improves dramatically for more recently Gavi-supported vaccines compared with less recently supported vaccines. Despite supply constraints, among the RI vaccines, IPV experienced the most rapid introduction across Gavi countries and achieved higher coverage in a shorter period of time, likely influenced by the unique global push and single dose regimen, which may not be applicable to many other vaccines (notwithstanding COVID-19 vaccines). Nonetheless, IPV demonstrates what is possible for country-level vaccine implementation timelines if there is a concerted ‘push’ by the global vaccine community, and could be considered a new benchmark for NVI uptake and introduction timelines.

### Limitations

This study had a number of important limitations. Sample sizes for country introduction and uptake were smaller for ‘newer’ vaccines, and these data may represent a subset of higher performing countries, potentially biasing timelines to appear more accelerated for these vaccines. A retrospective analysis that encompasses only vaccines that have already been introduced into all Gavi-supported countries may avoid this potential bias but would be limited in scope by the small number of Gavi-supported vaccines currently introduced into all Gavi-supported countries. By including vaccines in different stages of implementation, we allow for identification of opportunities to accelerate remaining NVIs during the implementation process; however, the majority of data points were for RI vaccines and findings may be less relevant for newer vaccines or those requiring new delivery platforms. We also do not analyse drivers of country timelines or individual country contexts to identify any common factors among accelerated timelines, nor do we make comparisons between Gavi-supported countries by regions, income categories or other archetypes, potentially limiting understanding of their influence. Lastly, Gavi-supported countries where sufficient data sharing agreements were not in place were not included in the study.

Our study also tied country coverage targets to a country’s own DTP3 level, which masks poor performance in countries with low DTP3 coverage rates, as these countries were considered having achieved target coverage after matching their existing DTP3 coverage level. While the immunisation coverage rate for the DTP3 increased from 68% in 2000 to 80% in 2016 across Gavi-supported countries, this improvement falls short of the 90% coverage target outlined in global goals. Treating the Gavi cohort as one unit also limits comparability between countries and masks disparities within countries.

### Potential to inform global health policy and practice

These findings are critical as Gavi, its partners and Gavi-supported countries focus on the dual goals of accelerating the pace of new introductions and improving uptake within and between Gavi-supported countries, both during and after the COVID-19 pandemic. Our findings can augment the global immunisation community’s understanding of how the Gavi partnership model has contributed to these goals, and the roles of different partners, such as WHO/SAGE, in helping to accelerate future introductions, especially for vaccines requiring new delivery platforms. This includes global partners closely coordinating on the various steps needed and working to complete various milestones in parallel or soon after a preceding one is completed, such as vaccine manufacturers being prepared to submit PQS dossiers as soon as they receive NRA licensure or the Gavi Board approving a new vaccine programme prior to first licensure. Measurement of milestones and standardisation of benchmarks for NVI may provide yet further guidance for Gavi, countries and donors in improving performance and shine light on bottlenecks that need further attention. Although some delays are unavoidable, such as unexpected manufacturing challenges or changes in government, some may be mitigated through increased global partner and country focus on NVIs, efforts to accelerate reporting of data that can lead to clearer guidelines, emphasis on demand generation and increased country readiness for NVIs. Focus on appropriate vaccine presentations to enable delivery and uptake in a diverse range of contexts will also become increasingly important with Gavi and Gavi-supported countries increasingly focused on immunising zero dose children or new and broader populations, particularly outside of the infant population.

### Implications for COVID-19 vaccine uptake

This analysis examined NVI and uptake timelines in the pre-pandemic era; however, key findings are readily relevant for Gavi-supported rollout of COVID-19 vaccines. The COVID-19 pandemic has achieved unprecedented political will and attention to accelerate every aspect of development and preparedness for COVID-19 NVIs facilitated by WHO’s Access to COVID-19 Tools (ACT) Accelerator and the COVAX Facility. Many of the factors identified in this paper correlated with accelerating NVIs are being realised in real time for COVID-19 vaccines. This includes achieving global-level VPD milestones such as Gavi Board approval for NVI support (through the COVAX Facility) prior to first NRA licensure, rapid first WHO PQ following first NRA licensure and Gavi application window opening rapidly following Gavi Board approval for funding support (through the COVAX Facility).^34^ In addition, the completion of the global-level VPD milestones by COVID-19 vaccines are already outpacing the timing benchmarks analysed in this paper, and NVI and scale-up timings are likely to also outpace those analysed in this paper. Furthermore, our findings indicate that that even in a supply-constrained environment, with concerted global support and rapid country application submissions, accelerated NVI and scale-up can be achieved across many Gavi-supported countries for both RI and campaign vaccines. While accelerated NVIs and uptake was not seen for the vaccine analysed in this paper (HPV) that required a new delivery platform, with the unprecedented global and national-level commitment and technical support in place and the rapid application submissions by countries for COVID-19 vaccines, the example of some of the other Gavi-supported vaccines that had global initiatives in place (IPV, MenA) lends evidence to rapid NVI and scale-up being possible even if there are supply constraints. This appears to already be happening with COVID-19 vaccines, as nearly 30 million doses have been sent to more than 50 COVAX-eligible countries at the time of paper submission, and even with the added challenge of reaching new and broader populations outside of traditional infant RI programmes and campaigns, vaccinations of these broader populations have begun.^35^

While it is unlikely this type of funding and effort could be sustained for other NVIs in Gavi-supported countries, there are important learnings from COVID-19 vaccines about reaching broader populations and delivery through different platforms that can inform NVIs going forward. This includes an understanding about how the COVID-19 NVI process leveraged the expertise of new stakeholders from EPI and other health programmes, civil society and communities. These learnings also include how regional and country partners can help plan and implement NVIs, mobilise resources and demand accountability, as well as how communication, coordination, use of data, demand generation and political will influence the ultimate timelines for action and uptake. Understanding the factors influencing this process may help in the development of new timelines that further enable acceleration and perhaps eliminate or condense steps that may result in unnecessary delays. Although a pandemic is clearly not comparable with the regular environment for NVIs, future analysis of factors that both enabled the accelerated introduction of COVID-19 vaccines while looking at any positive or negative impacts on supply, demand, and policies for other vaccines would help in establishing new metrics and milestones on which to benchmark future NVIs beyond the timelines established in this paper.

## Conclusion

Each VPD analysed in this study faced unique circumstances impacting its introduction timeline and pathway. While no simple conclusions can be made about what contributes to acceleration across the board, lessons can be learned from vaccines with short and historically long timelines that can be used by countries and the global immunisation community to accelerate future introductions regardless of delivery platform. Documentation of heterogeneity in performance by delivery platform and over time also highlights the need to continue to measure progress and learn from what works and what does not. This study also outlines what is possible for NVI and uptake rates, providing a baseline from which to improve further. Future monitoring of NVI and uptake timelines for COVID-19 vaccines into Gavi-supported countries can also contribute to understanding introduction timelines for new populations and delivery platforms.

## Data Availability

Data are available in a public, open access repository. Data are available on request. All data relevant to the study are included in the article or uploaded as online supplemental information. Much of the analysis was available through publicly accessible sources at World Health Organization. WHO/UNICEF Estimates of National Immunization Coverage for 1980–2016, 2017 (available from: http://www.who.int/immunization/monitoring_surveillance/data/en/), United Nations Department of Economic and Social Affairs – Population Division. World Population Prospects, the 2015 Revision, 2015 (available from: https://esa.un.org/unpd/wpp/Download/Standard/Population/).
