# Supplementary material for: Assessing vaccine introduction and uptake timelines in Gavi-supported countries: are introduction timelines accelerating across vaccine delivery platforms?
Source: BMJ Glob Health. 2021 May 27;6(5):e005032. doi: 10.1136/bmjgh-2021-005032 (PMC8162093; doi:10.1136/bmjgh-2021-005032)
Supplement: Supplementary data [file bmjgh-2021-005032supp001.pdf]

SUPPLEMENTAL TABLES

Supplemental Table 1: Vaccine products included in analysis

Vaccine Products by VPD & Supplier	Number of WHO PQ Vaccine Products (unique presentation, formulation, vial size, indication)
Pentavalent (n=25)	
Berna Biotech Korea Corporation	2
Bio Farma	2
Biological E Limited	6
GlaxoSmithKline	3
LG Life Sciences Ltd.	2
Panacea Biotec	2
Serum Institute of India LTD, India	6
Shantha Biotechnics Private Ltd.	2
Pneumococcal conjugate (n=4)	
GlaxoSmithKline	2
Pfizer / Wyeth	2*
Rotavirus (n=4)	
GlaxoSmithKline	2
Merck Sharp & Dohme Corp.	1
GlaxoSmithKline	1
HPV (n=3)	
GlaxoSmithKline	2
Merck Sharp & Dohme Corp.	1
Meningococcal A conjugate (n=2)	
Serum Institute of India LTD, India	2
Total	38

n = number of currently and previously prequalified vaccine products identified per VPD and one product not prequalified but introduced into two countries.
* We analyzed PCV-13 and PCV-7 (Pre-filled syringes, which were not prequalified). An additional 1-dose vial 7V was pre-qualified, but not introduced in any Gavi-supported countries and was not included in the study.

Supplemental Table 2: Gavi supported countries included in the analysis

Country Category	WHO Regions					
	AFRO (n=36)	AMRO (n=6)	EMRO (n=6)	EURO (n=6)	SEARO (n=9)	WPRO (n=6)
Gavi Graduating (UMIC + LMIC) (n=24)	Angola<br>Congo<br>Ghana<br>Nigeria	Bolivia<br>Cuba<br>Guyana<br>Honduras<br>Nicaragua		Armenia<br>Georgia<br>Moldova<br>Uzbekistan	Bhutan<br>Indonesia<br>Sri Lanka<br>Timor-Leste	Kiribati<br>Papua New Guinea<br>Solomon Islands<br>Vietnam
Gavi Intermediate (LMIC + LIC) (n=15)	Cameroon<br>Cote d'Ivoire<br>Lesotho<br>Mauritania<br>Sao Tome & Príncipe<br>Senegal<br>Zambia		Djibouti<br>Pakistan<br>South Sudan<br>Sudan<br>Yemen	Kyrgyzstan	India	Laos
Gavi Low Income (LMIC + LIC) (n=34)	Benin<br>Burkina Faso<br>Burundi<br>CAR<br>Chad<br>Comoros<br>DRC<br>Eritrea<br>Ethiopia<br>Gambia<br>Guinea<br>Guinea-Bissau<br>Kenya<br>Liberia<br>Madagascar	Haiti	Afghanistan	Tajikistan	Bangladesh<br>Myanmar<br>Nepal<br>North Korea	Cambodia

	Malawi<br>Mali<br>Mozambique<br>Niger<br>Rwanda<br>Sierra Leone<br>Tanzania<br>Togo<br>Uganda<br>Zimbabwe					

69 Gavi-supported countries are included in the analysis (68 of 72 countries eligible for Gavi Phase II support from 2007 to 2010, plus South Sudan). Gavi country category is based on 2015 Gavi co-financing policy; income category based on 2015 World Bank income classification.

Abbreviations: UMIC – upper middle-income country; LMIC – lower middle-income country; LIC – low income country; CAR – Central African Republic; DRC - Democratic Republic of Congo

Supplemental Table 3: List of milestones and data sources by VPD-level and country-level

Milestone	Data sources
**Global-level VPD Milestones**	
1st WHO-recognized NRA licensure per VPD	[1-13] Additional data provided via personal communications (Gavi, the Vaccine Alliance, 2016; Johnson & Johnson, 2016; Biological E Limited, 2016; Pfizer, 2016, Hib Initiative, 2016).
1st WHO PQ approval per VPD	[10] Additional data provided via personal communications (World Health Organization, 2016)
First SAGE recommendation	[14]
Gavi Board approval for support	Data provided via personal communications (Gavi,the Vaccine Alliance, 2016)
Gavi application window opened	Data provided via personal communications (Gavi,the Vaccine Alliance, 2016)
UNICEF tender issued	Data provided via personal communications (UNICEF, 2016)
1st Gavi-supported country introduction	Data provided via personal communications (Gavi,the Vaccine Alliance, 2016)
50% of target coverage (DTP3) reached across the Gavi cohort	[15 16]
100% of target coverage (DTP3) reached across the Gavi cohort	[15 16]
**Country Introduction Milestones**	
Gavi country NVI funding application submission	[17] Additional data provided via personal communications (Gavi,the Vaccine Alliance, 2016 & 2018)
Projected introduction date in Gavi country application	[17] Additional data provided via personal communications (Gavi,the Vaccine Alliance, 2016 & 2018)
Gavi country application approval	[18] Additional data provided via personal communications (Gavi,the Vaccine Alliance, 2016 & 2018)
Gavi vaccine introduction grant (VIG) disbursed to Gavi country	Data provided via personal communications (Gavi,the Vaccine Alliance, 2016 & 2018)
Vaccine introduction date in Gavi country	[17] Additional data provided via personal communications (Gavi,the Vaccine Alliance, 2016 & 2018)
50% of target coverage (DTP3) reached in Gavi country	[15]
100% of target coverage (DTP3) reached in Gavi country	[15]

Abbreviations: VPD – vaccine-preventable disease; NRA – national regulatory authority; PQ – prequalification; SAGE – Strategic Advisory Group of Experts on Immunization; DTP3 – third dose of diphtheria-tetanus-pertussis vaccine

Supplemental Table 4: Vaccine Products Analysis Results

A) List of milestones analyzed and data sources for vaccine products across all VPDs

Milestones	Data Source
1st WHO-recognized NRA licensure	[1-13] Additional data provided via personal communications (Gavi, the Vaccine Alliance, 2016; Johnson & Johnson, 2016; Biological E Limited, 2016; Pfizer, 2016, Hib Initiative, 2016).
WHO PQ dossier submission	Data provided via personal communications (World Health Organization, 2016)
WHO PQ approval	[10] Additional data provided via personal communications (World Health Organization, 2016)

B) Summary of median timelines across 5 VPDs and related vaccine products listed by most common order. VPDs include Penta, PCV (all), RVV, HPV, and MenA.

Starting Milestone	Ending Milestone	Median Metric (Range), Years[a]	IQR, Years	n
1st NRA licensure	WHO PQ dossier submission	0.4 (-0.9, 9.5)	0 .0 – 1.0	32
WHO PQ dossier submission	WHO PQ approval	1.1 (0.1, 3.0)	0.8 – 1.9	31
1st NRA licensure	WHO PQ approval	1.9 (0.1, 9.9)	1.3 – 3.1	38

[a] Times can be negative when the milestones occurred chronologically out of the specified expected order.

Supplemental Table 5: Analysis of individual VPD's milestone timelines

A) Timeline to first Gavi-supported introduction from first VPD level milestone by chronological order of milestones

VPD[a]	Mil. 1 to Mil. 2, Years	Mil. 2 to Mil. 3, Years	Mil. 3 to Mil. 4, Years	Mil. 4 to Mil. 5, Years	Mil. 5 to Intro, Years	Mil. 1 to Intro, Years
**Penta**	Lic. to SAGE<br>1.7	SAGE to PQ<br>0.1	PQ to Gavi<br>2.3	Gavi to UNICEF<br>0.3	UNICEF to Intro<br>1.1	Lic. to Intro<br>5.3
**PCV (all)**	Lic. to SAGE<br>5.9	SAGE to Gavi<br>0.8	Gavi to UNICEF<br>2.9	UNICEF to PQ<br>0.1	PQ to Intro<br>1.1	Lic. to Intro<br>10.8
**PCV10/ PCV13**	SAGE to Gavi<br>0.8	Gavi to Lic.<br>2.1	Lic. to UNICEF<br>0.8	UNICEF to PQ<br>0.1	PQ to Intro<br>1.1	SAGE to Intro<br>4.9
**RVV**	Lic. to Gavi<br>0.7	Gavi to PQ<br>0.2	PQ to SAGE<br>0.3	SAGE to UNICEF<br>3.7	UNICEF to Intro<br>0.4	Lic. to Intro<br>5.4
**HPV (demo)**	Lic. to SAGE<br>2.6	SAGE to PQ<br>0.4	PQ to Gavi<br>2.5	Gavi to UNICEF<br>1.0	UNICEF to Intro<br>0.5	Lic. to Intro<br>6.9
**MenA (C)**	SAGE to Gavi<br>6.1	Gavi to Lic.<br>1.2	Lic. to UNICEF<br>0.3	UNICEF to PQ<br>0.1	PQ to Intro<br>0.2	SAGE to Intro<br>7.9
**MenA (RI)**	SAGE to Gavi<br>6.1	Gavi to Lic.<br>6.1	Lic. to PQ<br>0.1	PQ to UNICEF<br>0.5	UNICEF to Intro<br>1.1	SAGE to Intro<br>13.8

[a]IPV excluded given skewed timelines as 1st NRA licensure occurred decades ago.

Key: Mil. – Milestone; Lic. – first NRA Licensure; SAGE: first SAGE recommendation; PQ – first WHO prequalification; Gavi – Gavi Board approval; UNICEF – UNICEF tender issued; Intro – first Gavi-supported country introduction

B) Timeline to first Gavi-supported introduction from each VPD level milestone

VPD[a]	Lic. to Intro, Years	PQ to Intro, Years	SAGE to Intro, Years	Gavi to Intro, Years	UNICEF to Intro, Years	No. of Mil. Prior to Lic.	No. of Mil. Prior to Gavi
**Penta**	5.3	3.6	3.7	1.3	1.1	0	3
**PCV (all)**	10.8	1.1	4.9	4.1	1.2	0	3
**PCV10/ PCV13**	2.0	1.1	4.9	4.1	1.2	2	1
**RVV**	5.4	4.4	4.1	4.7	0.4	0	1
**HPV (demo)**	6.9	4.0	4.3	1.5	0.5	0	3
**MenA (C)**	0.6	0.2	7.8	1.8	0.3	2	1
**MenA (RI)**	1.7	1.6	13.8	7.7	1.1	2	1

[a]IPV excluded as it was not included in the VPD-level analysis.

Key: Mil. – Milestone; Lic. – first NRA Licensure; SAGE: first SAGE recommendation; PQ – first WHO prequalification; Gavi – Gavi Board approval; UNICEF – UNICEF tender issued; Intro – first Gavi-supported country introduction

Supplemental Table 6: Country introduction decision making (represented by submission of Gavi funding application) timelines by VPD

Time (years) from below global-level VPD milestone to 50% and 100% of country submissions of Gavi NVI funding applications							
**VPD[a]**	**Gavi application window opening**	**First SAGE Rec. per VPD**	**First SAGE Rec. applicable to all Gavi-supported countries?**	**First SAGE Rec. applicable to all Gavi countries**	**First SAGE Rec. applicable to all Gavi countries issued prior to application window opening?**	**% of country NVI funding apps submitted at time of SAGE Rec applicable to all Gavi countries**	n[b]
**Time (years) to 50% of Gavi country applications submitted**							
Penta	7.0	9.1	No	1.3	No	29%	65[d]
PCV (all)	3.8	5.3	No	4.3	Yes	0%	69
RVV	6.2	6.3	No	5.2	Yes	0%	69
MenA (C)	4.9	11.0	No	2.4	No	11%	26
MenA RI[c]	-	-	No	-	Yes	0%	69
HPV (demo)[c]	-	7.7	Yes	3.7	Yes	0%	69
HPV (national)[c]	-	-	Yes	-	Yes	0%	69
IPV	0.6	-[e]	-[e]	-[e]	-[e]	-[e]	68
**Time (years) to 100% of Gavi country applications submitted**						**N/A**	
Pentavalent	12.3	14.5	No	6.7	No		65[d]
PCV[c]	-	-	No	-	Yes		69
RVV[c]	-	-	No	-	Yes		69
MenA (C)[c]	-	-	No	-	No		26
MenA RI[c]	-	-	No	-	Yes		69
HPV (demo)[c]	-	-	Yes	-	Yes		69
HPV (national)[c]	-	-	Yes	-	Yes		69
IPV	1.0	-[e]	-[e]	-[e]	-[e]		68

[a]Calculated for vaccines delivered as part of the infant routine immunization programs and campaigns only; HPV demonstration projects excluded. [b]n = number of countries; excludes countries that had already introduced the vaccine, were no longer eligible for Gavi support at time of Board decision, or for which no data was available. Gavi MenA support covers 26 'meningitis belt' countries. [c]Number of country applications have not yet reached this milestone at time of data collection. [d]All 69 Gavi-supported countries included in this analysis were eligible to apply and introduce pentavalent; information was not available on all applications [e]IPV excluded from analysis of SAGE recommendation timelines as IPV was not included in the VPD-level analysis
Abbreviations: VPD – vaccine-preventable diseases

SUPPLEMENTAL FIGURES

Supplemental Figure 1: The hypothesized full pathway from first NRA licensure to first country introduction and country uptake – VPD-level milestones (A) and the hypothesized full introduction pathway at the country level –country-level milestones (B)

A.

B.

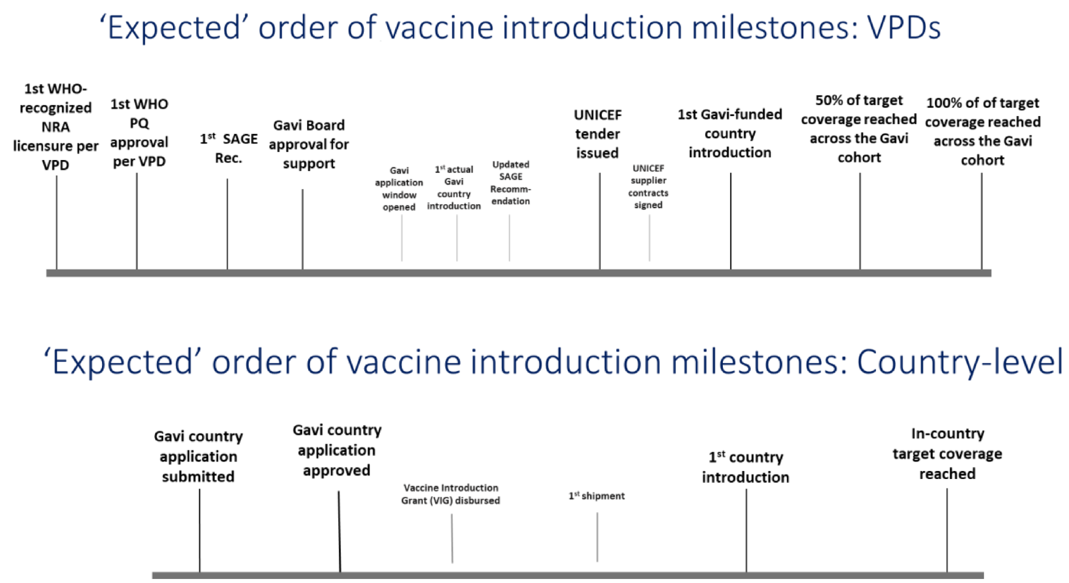

Key: NRA: National regulatory authority; WHO PQ: World Health Organization Prequalification; VPD: vaccine preventable disease; target coverage defined as 90% of DTP3 coverage in the country that year.

Supplemental Figure 2: Time from first chronological VPD milestone to first Gavi-supported country introduction

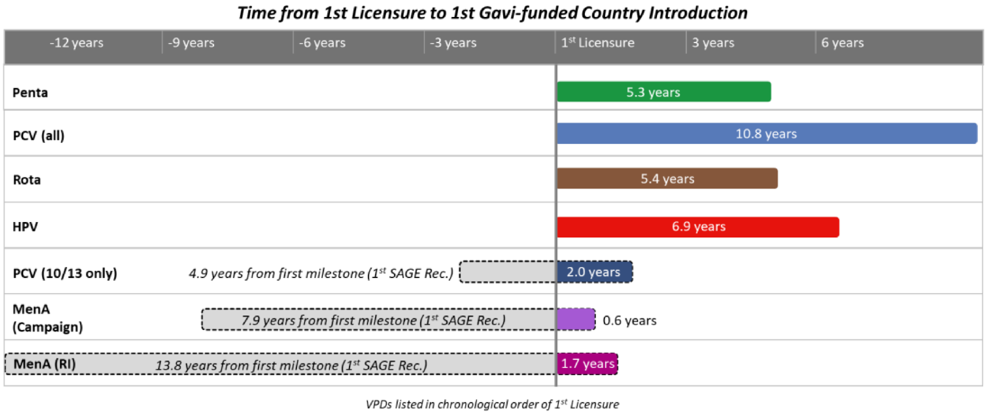

Time from first chronological VPD milestone to first Gavi-supported country introduction, with noting of how timelines compare starting from first licensure. The quickest introduction into Gavi countries following first vaccine product licensure had the first VPD milestone occur prior to first licensure (depicted in grey color).

Supplemental Figure 3: Measures of delay in NVIs across Gavi-supported countries by VPD

A. Frequency of NVI Timelines for Gavi-Supported Countries following Gavi Application Approval Across 69 Gavi-supported countries and 5 VPDs

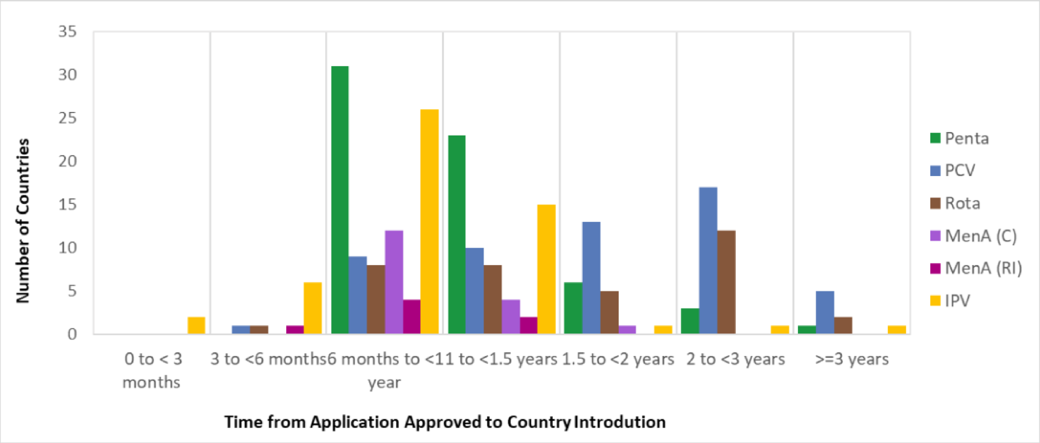

** HPV excluded given application approval process differed from other VPDs during part of the time frame of analysis.*

Timelines for time from Gavi application approval to date of NVI in a Gavi-supported country.

B. Frequency of Delays of Gavi-Supported NVIs Compared to Proposed Introduction Dates Across 69 Gavi-supported countries and 6 VPDs

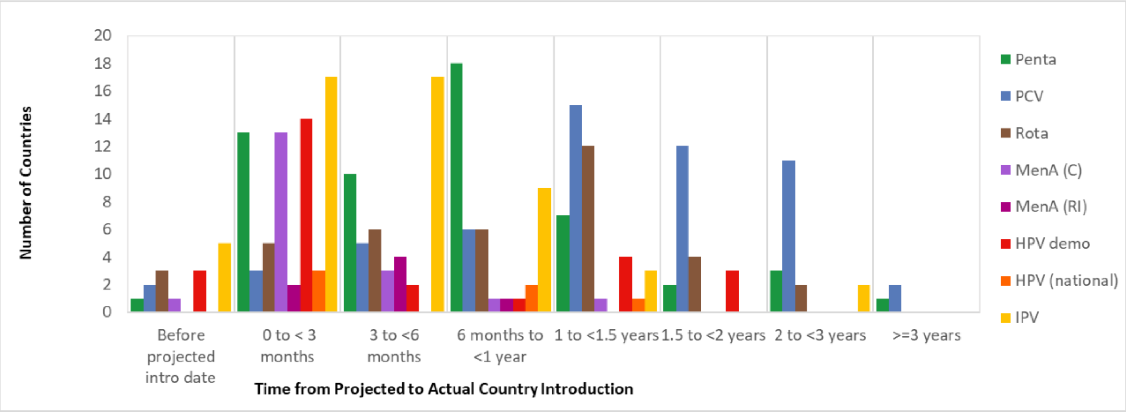

Timelines for delay in projected NVI date as named in the Gavi application compared to the data of actual first introduction in a Gavi-supported country.

SUPPLEMENTARY MATERIAL REFERENCES

1. World Health Organization. National regulatory authorities (NRAs) and National Control Laboratories (NCLs) in countries producing vaccines prequalified for purchase by UN agencies, 2012.
2. Crucell N.V. Crucell Announces Product Approval in Korea for Quinvaxem Vaccine.: DGNews, 2006.
3. Medicines Evaluation Board - The Netherlands. Medicines Information Bank: Medicines Evaluation Board - The Netherlands; 2015 [Available from: http://db.cbg-meb.nl/ords/f?p=111:1:0:::SESSION:P0_DOMAIN,P0_LANG:H,EN accessed December 16, 2015.
4. Thai Bureau of Drug Control. Poliorix Summary of Product Characteristics, 2015. [Available from: http://drug.fda.moph.go.th/zone_search/files/Polirix _2C 35_47_N.pdf accessed December 16, 2015.
5. Danish Health and Medicines Authority. Produktresume for Poliovaccine "SSI" 2012 [Available from: http://www.produktresume.dk/docushare/dsweb/GetRendition/Document-24965/html#bmk31 accessed December 16, 2015.
6. Meningitis Vaccine Project. Regulatory and prequalification pathways 2014 [Available from: http://www.meningvax.org/regulatory-prequalification.php accessed December 17, 2015.
7. PT Indofarma (Persero) Tbk. Annual Report 2013, 2013. [Available from: http://www.indonesia-investments.com/upload/bedrijfsprofiel/2018/indofarma-annual-report-2013-inaf-company-profile-indonesia-investments.pdf accessed January 28, 2016.
8. Summary of Product Characteristic (SmPC): Central Drug Standard Control Organization; Government of India; 2015 [Available from: http://www.cdsco.nic.in/Forms/list.aspx?lid=1993 accessed December 16, 2015.
9. Vaccines Registered: Central Drug Standard Control Organization; Government of India; 2012 [Available from: http://www.cdsco.nic.in/Forms/SearchMore.aspx?Id=1&lid=1601 accessed December 16, 2015.
10. World Health Organization. WHO prequalified vaccines: World Health Organization; 2015 [Available from: http://www.who.int/immunization_standards/vaccine_quality/PQ_vaccine_list_en/en/ accessed October 27, 2015.
11. Health Canada - Health Products and Food Branch. Summary Basis of Decision (SBD) for SYNFLORIX, 2009. [Available from: http://www.hc-sc.gc.ca/dhp-mps/alt_formats/pdf/prodpharma/sbd-smd/drug-med/sbd_smd_2009_synflorix_119056-eng.pdf accessed December 16, 2015.
12. U.S. Fod and Drug Administration. Approved Products - Complete List of Vaccines Licensed for Immunization and Distribution in the US: U.S. Food and Drug Administration; 2015 [Available from: http://www.fda.gov/BiologicsBloodVaccines/Vaccines/ApprovedProducts/UCM093833 accessed December 3, 2015.
13. Australian Government Department of Health Therapeutics Goods Administration.. Australian Register of Therapeutic Goods: Australian Government Department of Health Therapeutics Goods Administration; 2015 [Available from: https://www.ebs.tga.gov.au accessed December 16, 2015.
14. World Health Organization. SAGE reports from previous meetings. WHO Weekly Epidemiological Record 1998 - 2016. 2016 [Available from: http://www.who.int/immunization/sage_conclusions/en/ accessed February 26, 2016.
15. World Health Organization. WHO/UNICEF Estimates of National Immunization Coverage for 1980-2016, 2017. [Available from: http://www.who.int/immunization/monitoring_surveillance/data/en/ accessed October 16, 2017.
16. United Nations Department of Economic and Social Affairs - Population Division. World Population Prospects, the 2015 Revision, 2015. [Available from: https://esa.un.org/unpd/wpp/Download/Standard/Population/ accessed January 18, 2016.

17. Gavi, the Vaccine Alliance. Country Hub 2016 [Available from: http://www.gavi.org/country/ accessed March 21, 2016.
18. Gavi, the Vaccine Alliance. Board minutes & executive committee minutes 2016 [Available from: http://www.gavi.org/about/governance/gavi-board/minutes/ accessed March 21, 2016.
